# Multisource Deep Transfer Learning Based on Balanced Distribution Adaptation

**DOI:** 10.1155/2022/6915216

**Published:** 2022-04-18

**Authors:** Peng Gao, Jingmei Li, Guodong Zhao, Changhong Ding

**Affiliations:** ^1^College of Computer Science and Technology, Harbin Engineering University, Harbin, China; ^2^Converged Media Technology Department, Heilongjiang Broadcasting Station, Harbin, China; ^3^Heilongjiang University of Chinese Medicine, Harbin, China

## Abstract

The current traditional unsupervised transfer learning assumes that the sample is collected from a single domain. From the aspect of practical application, the sample from a single-source domain is often not enough. In most cases, we usually collect labeled data from multiple domains. In recent years, multisource unsupervised transfer learning with deep learning has focused on aligning in the common feature space and then seeking to minimize the distribution difference between the source and target domains, such as marginal distribution, conditional distribution, or both. Moreover, conditional distribution and marginal distribution are often treated equally, which will lead to poor performance in practical applications. The existing algorithms that consider balanced distribution are often based on a single-source domain. To solve the above-mentioned problems, we propose a multisource transfer learning algorithm based on distribution adaptation. This algorithm considers adjusting the weights of two distributions to solve the problem of distribution adaptation in multisource transfer learning. A large number of experiments have shown that our method MTLBDA has achieved significant results in popular image classification datasets such as Office-31.

## 1. Introduction

Machine learning can achieve good results in computer vision, and it is often based on the following assumptions: there are enough data samples in the training dataset and a high-precision classifier; the training data and testing data come from the same feature space and the same distribution. For a new domain, it is often difficult to obtain enough data labels. In this case, transfer learning [1] is a promising method that transfers knowledge from the source domain to the target domain. At the same time, the development of deep learning has accelerated the technical level of transfer learning models. Transfer learning usually assumes that training and testing data come from similar but different distributions [2]. For example, the object that takes a photo under different angles, backgrounds, and lighting may get different marginal condition distributions. The existing transfer learning methods mainly focus on distributed adaptation by observing and reducing the difference between each domain through joint distribution adaptation. For example, several unsupervised transfer learning methods [3–5] use maximum mean discrepancy in the neural network to reduce the domain difference; other models introduce different learning modes to align the source and target domains, including aligning second-order correlation [6, 7].

In recent years, most unsupervised transfer learning algorithms have focused on single-source unsupervised transfer learning problems, which are training samples that come from a single-source domain. In previous research, the work focused on estimating the sample's weight, which is the ratio of the source domain and the target domain [8–11]. In addition, the manifold learning method is used to sample a high-dimensional space and map it to a low-dimensional manifold space to make sure that the subspace of the source domain and the target domain comes closer. Some single-source transfer learning algorithms map the data of two domains to a common feature space and describe the invariant features of the source and target domains by minimizing the difference in domain distribution [6, 12–14]. Long [15], Hou [16], and Hashemi [17] had also proposed many joint distribution adaptive methods to solve the difference of distribution between the source domain and the target domain. In recent years, many deep transfer learning algorithms were proposed to solve the problem of data distribution adaptation. Tzeng et al. proposed DDC (deep domain confusion) [18], and Long [12] et al. proposed the DAN (deep adaptation network) to solve the problem of marginal distribution adaptation. Zhu [19] et al. proposed the DSAN (deep dynamic adaptation network), and Wang [20] proposed the DDAN (deep dynamic adaptation network) to solve the problem of jointly distributed adaptation.

However, in practical work, we often face multiple source domains, so it is more feasible to study the migration of multiple source domains, and it is also more meaningful in practice. For multisource transfer learning, a common simple idea is to combine all source domains into a new source domain and then use the single-source transfer learning algorithm to classify the target domain data. Due to the expansion of the dataset, these methods may yield better results. However, in practical applications, because of the large differences in the distribution of each domain, this type of method does not yield good results. Therefore, we need to find a better way to utilize data from multiple source domains.

With the rapid development of deep learning, there are many studies on transfer learning based on deep learning. Zhao [21] et al. proposed a multidomain adversarial network, which aligns the distribution of features in each source domain and target domain through multiple domain discriminators; Xu [22] et al. proposed a deep cocktail network. A separate domain discriminator and a classifier are designed for each source domain and target domain. The current deep multisource transfer learning algorithms often have the following two problems: 1. They first map the source domain samples and target domain samples to the same common feature space, but even for a single-source domain. It is also difficult to learn the same characteristics as those of the target domain. Moreover, in multiple source domains, their data samples are likely to cross, which leads to a reduced effect of feature alignment. 2. At present, the studies often consider only the marginal probabilities or conditional probabilities for the distribution of the source domain and the target domain. Current algorithms often adjust the marginal probability first and then adjust the conditional probability. The relationship between them is not fully utilized.

In this article, we combine the advantages of balancing distribution, convolutional neural networks, and multisource transfer learning, and then a new multisource transfer learning algorithm based on balanced distribution adaptation—MTLBDA—is proposed, which first maps multiple source domains and target domains to the same subspace and then aligns the features of multiple source domains and target domains. Then, according to the balanced distribution adaptation, the effect of the category in each source domain and target domain is decreased, and the difference between the marginal probability distribution and the conditional probability distribution in each source domain and target domain is reduced. Then the convolutional neural network is used as the classifier for each source domain and target domain to complete the task of classification. Finally, we generate a regularization term for the classifier of each source domain, which is weighted to prevent overfitting of the model.

Compared with the previous work, the contributions of this work include the following:

1. A new multisource transfer learning algorithm named MTLBDA is proposed, which balances the difference between conditional probability distribution and marginal probability distribution to improve the classification effect. This method first maps all domains to the same feature space and then reduces the difference between the marginal probability distribution and conditional probability distribution with maximum mean discrepancy and adds a separate regularization term to the convolutional neural networks on this basis.
2. For multisource domain samples, the conditional probability distribution and marginal probability distribution are considered. This method can adjust the category adaptation of the multisource domain and target domain.
3. Multiple source domains provide more knowledge on the learning tasks of the target domain. Compared with the trained classifier set and the independent classifier, the trained classifier set system has a better prediction effect and more stability.
4. Experiments on real datasets show that the proposed algorithm is superior to or at least comparable to advanced benchmark algorithms in classification accuracy.

The rest of the paper is arranged as follows: Section 2 reviews the work related to multisource transfer learning and joint distribution adaptation.  Section 3 proposes multisource deep transfer learning based on balanced distribution adaptation.  Section 4 verifies the effectiveness of the algorithm on the SVHN dataset, USPS dataset, MINIST dataset, Office-31 dataset, and DomainNet dataset.  Section 5 summarizes the main work of this paper.

## 2. Problem definition

### 2.1. Joint Distribution Adaptation

A domain often has two probability distributions: one is marginal probability, and the other is conditional probability. Long [21] gave the hypothesis of joint distribution adaptation, whose purpose is to reduce the distance of joint probability distribution between the source domain and the target domain. Current research on joint distribution includes domain invariant clustering [16], increasing structural consistency [17], target optimization [23], and so on. Wang [20] proposed a dynamic balance adaptive algorithm, which pointed out that marginal distribution adaptation and conditional distribution adaptation are not equally important. However, these joint distribution adaptations are often used in the field of single-source transfer learning, and they have not played a role in the field of multisource transfer learning.

### 2.2. Multisource Transfer Learning

**Figure 1:**
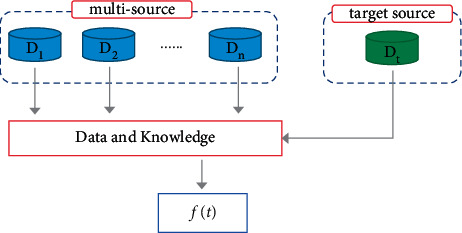
Multisource transfer learning.

Multisource transfer learning (as shown in Figure 1) as a research direction of transfer learning has essential practical value. In the process of real life and practical application, there are often multiple source domains. Although each source domain has a different similarity to the target, these source domains can still be used for knowledge transfer. Moreover, multisource transfer learning contains more knowledge, which can make the effect of the model better. At the same time, transfer learning also has a theoretical basis. Crammer [24] first proposed the expected loss boundary condition of multisource transfer learning. Later, Mansour [25] proved that the distribution weighted combination rule can reduce the instantaneous function between the source domain and the target domain. Ben-David [26] gave two learning boundaries for minimizing empirical risk by introducing the distance between the target domain and the source domain.

In recent years, a lot of work was centered around multisource transfer learning and deep learning. Xu [22] proposed the deep cocktail network (DCTN), which uses a single domain discriminator and a classifier for each source domain and target domain. The domain discriminator is used to align the feature distribution, and the classifier outputs the predicted probability distribution. Based on the output of the domain discriminator, the DCTN designed a method of voting by multiple classifiers. Peng [27] proposed a moment matching multisource domain adaptation (M^3^SDA) method, which not only considers the alignment between the source domain and the target domain but also aligns the feature distribution of different source domains. Zhu [28] et al. proposed a framework named aligning domain-specific distribution and classifier for cross-domain classification from multiple sources (MFSAN). However, the current deep multisource transfer learning algorithms often only consider the marginal probability distributions or consider the marginal probability distribution and the conditional probability distribution separately. In this paper, multisource transfer learning based on balanced distribution adaptation, which considers the joint probability distribution to improve the accuracy of the algorithm, is proposed.

Problem.

In multisource transfer learning, there are *N* source domains, and their labeled sample data can be represented as *X*^*s*_*i*_^={(*x*_*j*_^*s*_*i*_^, *y*_*j*_^*s*_*i*_^)}_*j*=1}_^*N*_*i*_^, where {(*x*_*j*_^*s*_*i*_^)}_*j*=1_^*N*_*i*_^ represents the *i*-th sample data in the *j*-th source domain and {(*y*_*j*_^*s*_*i*_^)}_*j*=1_^*N*_*i*_^ represents the *i*-th source domain in the *j*-th source domain. The joint probability distribution of *N* different domains can be expressed as {*P*^*s*_*i*_^(*x*, *y*)}_*i*=1_^*N*_*i*_^ , where the marginal probability can be expressed as {*P*^*s*_*i*_^(*x*)}_*i*=1_^*N*_*i*_^ and the conditional probability can be expressed as {*P*^*s*_*i*_^(*y*|*x*)}_*i*=1_^*N*_*i*_^. Similarly, we give the definition of the target domain; the sample of the target domain can be expressed as *X*^*t*^={(*x*_*j*_^*t*^)}_*j*=1_^*N*_*t*_^, and the probability distribution can be expressed as *P*^*t*^(*x*, *y*).

In recent years, some papers had defined the objective function of multisource deep transfer learning. They first map all domains to the same target space and then use the common domain invariant representation in the common feature space for learning all domains. Zhu [28] et al. gave a definition of the loss function:

(1) $$\begin{array}{c} \underset{F,C}{\min}\sum_{i=1}^{N}{Ε}_{x∼X_{s_{i}}}J\left(C_{i}H_{i}\left(F\left(x_{j}^{s_{i}}\right)\right),y_{j}^{s_{i}}\right)+\lambda \sum_{i=1}^{N}\overset{\wedge }{D}H_{i}\left(F\left(X_{s_{i}}\right),F\left(X_{t}\right)\right)+\gamma L_{\text{disc}}. \end{array}$$

The first term represents the loss of the classification function, the general classification loss is the cross-entropy loss, and the second term represents the statistical measurement of the source domain and the target domain. Nowadays, the commonly used metrics are MMD [15], reference loss [29], CORAL loss [12], and confusion loss [13, 14]. Zhu et al. defines CORAL loss as a specific difference loss. The common problem of these methods is that they only use MMD to calculate the marginal distribution difference between the source domain and the target domain without considering the influence of conditional probabilities on the model. Zhao [30]'s paper published on ICML2019 proved theoretically that reducing the marginal distribution difference between the source domain and the target domain is not enough. At the same time, Wang's paper also pointed out that equal consideration of marginal distribution and conditional probability distribution is not enough. Therefore, we propose a multisource deep transfer learning algorithm based on balanced distribution adaptation to solve these problems.

Similar to other multisource transfer learning algorithms, we first map multiple source domains and target domains to the same subspace, and then we align the marginal probability distribution and conditional probability distribution of each source domain and target domain. Of course, the best way is to tune the convolutional neural network for each pair of source and target domains. However, from a practical point of view, the amount of calculation in this method is very large, so we use shared weights to solve this problem. Finally, we add a specific regularization term to realize the problem of individual network tuning.

## 3. Multisource Deep Transfer Learning Based on Balanced Distribution Adaptation

To solve the impact of category imbalance on the existing multisource transfer learning algorithm, in this chapter, we introduce a multisource transfer learning algorithm based on distribution balance. We use the general regularization item proposed in [28] to replace the classification selector to output the final classification result.

Algorithm structure. Our algorithm structure contains three parts—a common feature selector, a distribution balancer, and a regularizer—as shown in the figure.

*Common feature extractor*: We propose a common subnet *f*(·) to extract the common representation of all domains, which maps images from the original feature space to a common feature space.

*Domain-specific distribution balancer*: We design a distribution balancer for each source domain and target domain data, given a set of images *x*_*j*_^*s*^ from the source domain *X*^*s*^={(*x*_*j*_^*s*^, *y*_*j*_^*s*^)} and a set of images *x*^*t*^ from the target domain {(*x*_*j*_^*t*^)}. The features of these specific fields are mapped to the same feature space through a common feature extractor, specifically expressed as the source domain mapping feature *f*(*x*_*j*_^*s*^) and target domain mapping characteristics *f*(*x*^*t*^). Hence, we can get *N* independently distributed balancers *b*(·) corresponding to specific source domains {(*x*_*j*_^*s*^, *y*_*j*_^*s*^)}.

The class balancer we proposed is a domain-specific feature extractor. Generally, people use MMD, CORAL, adversarial, and other methods as feature extractors, but they often only consider one distribution. To balance the categories, we use the BDA algorithm proposed by Wang Jindong [20] while considering conditional distribution, marginal distribution, and multiclass balance as the distribution balancers. We use a convolutional neural network as our classifier, and we define *C*_*i*_ as the classifier of *N* source domains. Based on experience, our loss is classified as cross-entropy loss, and the loss function is denoted as *J*(·, ·).

*Domain-specific regularization term*: Based on the behavior regularization proposed in the literature, for the source domain *i*, we give the regularization term *ℜ*(*w*, *w*^*∗*^, *x*_*j*_, *y*_*j*_), where **w** is the *d*-dimensional parameter vector containing all *d* parameters of the target domain under the convolutional neural network. **w**^*∗*^ is the parameter vector of the source domain. It is harder to calculate all parameters for each domain, so we share the parameters of the first *n* − 3 layers.

**Figure 2:**
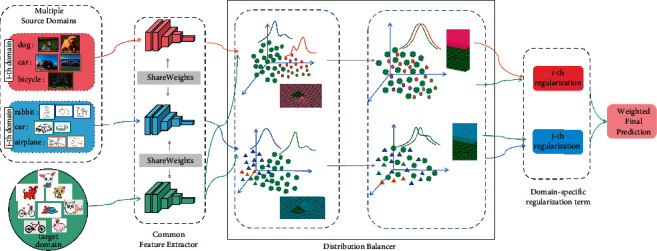
The framework of multisource deep transfer learning based on balanced distribution adaptation (MTLBDA). Our model consists of three components: (i) a common feature extractor, (ii) a domain-specific distribution balancer, and (iii) a domain-specific regularization term.

Objective function:

According to Figure 2, we define the final objective function of the algorithm as

(2) $$\begin{array}{c} L=L_{cls}+Lb_{bda}+Lr_{reg}. \end{array}$$

The classification loss *L*b_*cls*_ is the loss caused by a specific domain classifier, and in Figure 2, we can see that the variable *x*_*j*_ in the source domain *i* undergoes a three-step transformation: first, *F*(*x*_*j*_^*s*_*i*_^) is obtained through the public feature extractor; then *B*_*i*_(*F*(*x*_*j*_^*s*_*i*_^)) is obtained through the class balancer; finally, *C*_*i*_(*B*_*i*_(*F*(*x*_*j*_^*s*_*i*_^))) is obtained through the CNN classification. The final classification loss is

(3) $$\begin{array}{c} L_{cls}=\sum_{i=1}^{N}{Ε}_{x∼X^{s_{i}}}J\left(C_{i}B_{i}\left(F\left(x_{j}^{s_{i}}\right)\right),y_{j}^{s_{i}}\right). \end{array}$$

The balance loss *L*b_*bda*_ is a specific domain balancer loss, and we follow the concept of single-source domain distribution balancers according to Wang et al. The algorithm considers the conditional probability and marginal probability distribution of the source domains and target domains at the same time. In particular, due to the inability to obtain the label of the target domain, we have no way of estimating the conditional probability distribution. Therefore, we use the proof given in [31]; when there are enough label samples, we can use the conditional distribution *P*(*x*_*t*_|*y*_*t*_) of the class to approximately match the conditional distribution *P*(*y*_*t*_|*x*_*t*_). In calculating the conditional distribution *P*(*x*_*t*_|*y*_*t*_) of the class, we first use the specific domain classifier to label the target domain data samples to form the prelabels of the target domain samples.

(4) $$\begin{array}{c} D\left(D_{s},D_{t}\right)\approx \left(1-\mu \right)D\left(P\left(x_{s}\right),P\left(x_{t}\right)\right)+\mu D\left(P\left(y_{s}|x_{s}\right),P\left(y_{t}|x_{t}\right)\right). \end{array}$$

*μ* ∈ [0,1] is the balance factor. When *μ*⟶0, the marginal distribution is more important, and when *μ*⟶1, the conditional probability is more important. To calculate the marginal probability and conditional probability, according to MMD and TCA [32], we can define the specific domain balancer estimation empirically.

(5) $$\begin{array}{c} {\overset{\wedge }{D}}_{H}\left(p,q\right)={\left\|\frac{1}{n_{\text{s}}}\sum_{x_{a}\in X^{s}}\phi \left(x_{a}\right)-\frac{1}{n_{t}}\sum_{x_{b}\in X^{t}}\phi \left(x_{b}\right)\right\|}_{H}^{2},\\ \overset{\wedge }{D^{\text{cn}}}\left(p_{t},q_{t}\right)=\sum_{c=1}^{C}{\left\|\frac{1}{n_{\text{c}}}\sum_{x_{a}\in X_{\left(c\right)}^{s}}\phi \left(x_{a}\right)-\frac{1}{m_{c}}\sum_{x_{b}\in X_{\left(c\right)}^{t}}\phi \left(x_{b}\right)\right\|}_{H}^{2}. \end{array}$$

For the *i*-th source domain, the squared distance between the empirical kernel average embeddings is obtained from the empirical estimation of MMD.

(6) $$\begin{array}{c} \overset{\wedge }{D^{B}}=\left(1-\mu_{i}\right){\left\|\frac{1}{n_{\text{s}}}\sum_{x_{a}\in X^{s}}\phi \left(x_{a}\right)-\frac{1}{n_{t}}\sum_{x_{b}\in X^{t}}\phi \left(x_{b}\right)\right\|}_{H}^{2}+\mu_{i}\sum_{c=1}^{C}{\left\|\frac{1}{n_{\text{c}}}\sum_{x_{a}\in X_{\left(c\right)}^{s}}\phi \left(x_{a}\right)-\frac{1}{m_{c}}\sum_{x_{b}\in X_{\left(c\right)}^{t}}\phi \left(x_{b}\right)\right\|}_{H}^{2}. \end{array}$$

We define formula (6) as the estimation of the difference between the source domain and the target domain. Therefore, the balance loss is defined as follows:

(7) $$\begin{array}{c} L_{bda}=\sum_{i=1}^{N}\hat{D}\left(B_{i}\left(F\left(X^{s_{i}}\right),F\left(X^{t}\right)\right)\right). \end{array}$$

We define the regularization term for a specific domain *i* according to the behavior regularization term proposed in [33] as follows:

(8) $$\begin{array}{c} {ℜ}^{i}\left(w,w^{*},x_{j},y_{j}\right)=\sum_{j=1}^{n}\Omega \left(w,w^{*},x_{j},y_{j}\right),\\ \Omega \left(w,w^{*},x_{j},y_{j}\right)=\alpha \cdot \Omega^{\prime }\left(w,w^{*},x_{j},y_{j}\right)+\beta \cdot L^{2}\left(w,w^{*}\right),\\ \Omega^{\prime }\left(w,w^{*},x_{j},y_{j}\right)=\sum_{k=1}^{N}{\text{W}}_{k}\left(w^{*},x_{j},y_{j}\right)\cdot {\left\|FM_{k}\left(w,x_{j}\right)-FM_{k}\left(w^{*},x_{j}\right)\right\|}_{2}^{2}. \end{array}$$

*W*_*k*_(**w**^*∗*^, *x*_*j*_, *y*_*j*_) refers to the weight assigned to the j-th image in the k-th layer of the network, *FM*_*k*_(**w**, *x*_*j*_) · *FM*_*k*_(**w**^*∗*^, *x*_*j*_) refers to the difference in the characteristics of the two images, ‖·‖_2_ indicates their Euclidean distance, and *L*^2^(**w**, **w**^*∗*^) represents the *L*^2^ regularization term of **w** and **w**^*∗*^. In order to reduce calculation, *k*={*n*, *n* − 1, *n* − 2}. Collecting the regularization terms of multiple source domains, we define the regularization loss as

(9) $$\begin{array}{c} L_{reg}=ℜ\left(w,w^{*},x_{j},y_{j}\right)=\sum_{i=1}^{N}\gamma_{i}\cdot {ℜ}^{i}\left(w,w^{*},x_{j},y_{j}\right). \end{array}$$

*γ* ∈ [0,1] is the value of the regularization term ranging from 0 to 1, and its selected value is defined according to the subsequent selector.

Final objective function:

(10) $$\begin{array}{c} L=L_{cls}+Lb_{bda}+Lr_{reg},\\ L=\sum_{i=1}^{N}{Ε}_{x∼X^{s_{i}}}JC_{i}\left(B_{i}\left(F\left(x_{j}^{s_{i}}\right)\right.,y_{j}^{s_{i}}\right)\\ \;+\sum_{i=1}^{N}\hat{D}\left(B_{i}\left(F\left(X^{s_{i}}\right),F\left(X^{t}\right)\right)\right)\\ \;+\sum_{i=1}^{N}\gamma_{i}\cdot {ℜ}^{i}\left(w,w^{*},x_{j},y_{j}\right),\\ L=\sum_{i=1}^{N}\left({Ε}_{x∼X^{s_{i}}}J\left(C_{i}\left(B_{i}\left(F\left(x_{j}^{s_{i}}\right)\right)\right.,y_{j}^{s_{i}}\right)+\hat{D}\left(B_{i}\left(F\left(X^{s_{i}}\right),F\left(X^{t}\right)\right)\right)+\gamma_{i}\cdot {ℜ}^{i}\left(w,w^{*},x_{j},y_{j}\right.\right). \end{array}$$

In summary, the specific process steps of the MTLBDA algorithm are shown in Table 1.

## 4. Experimental Results

To test the effectiveness and generalization of the MTLBDA algorithm, we test it on two types of image datasets. The first type is a digital classification dataset including the SVHN [34] dataset, USPS [35] dataset, and MNIST [36] dataset. The second category is of image classification datasets including the Office-31 [37] dataset, Caltech [38] dataset, and DomainNet [24] dataset.

The experiment will compare single-source transfer learning algorithms DAN, DANN, BDA, and DDAN, and multisource transfer learning algorithms DCTN, MFSAN, and M^3^SDA.

For fairness of the experiment, a 5-fold cross-validation strategy is selected for all experiments, and the experiments of this strategy are repeated twice to obtain the final comparison result. In the experiment, we use the average classification accuracy [39, 40] and recall rate of each algorithm after running it for 10 times as the evaluation criteria. The recall rate reflects how many positive examples in the sample are predicted correctly. The forms of expression of classification accuracy and recall are defined as follows:

  Classification accuracy: Accuracy=|*x* : *x* ∈ *X*∧*f*(*x*)=*y*(*x*)|/|*x* : *x* ∈ *X*|.
  Recall rate: *R*=*FP*/*TP*+*FN* × 100%.

Among them, TP represents the number of positive samples that are correctly classified as positive, FP represents the number of negative samples that are incorrectly classified as positive, TN represents the number of negative samples that are correctly classified as negative, and FN represents the number of positive samples that are incorrectly classified as negative.

X represents the target domain number test dataset, *f*(*x*) is the sample *x*-class label predicted by the classifier, and *y*(*x*) is the reality-class label of sample *x*.

### 4.1. Digital Classification Dataset

#### 4.1.1. Dataset Introduction

**Figure 3:**
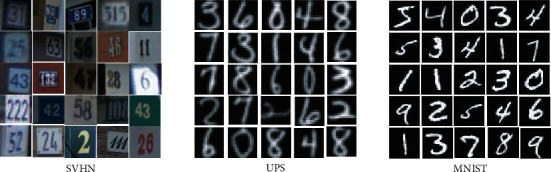
Example of USPS, MNIST, and SVHN pictures.

Both the USPS dataset and the MNIST dataset contain handwritten digits “0”–“9”; the former is composed of 9298 16 × 16 images, and the latter is composed of 70,000 28 × 28 images. The street view house number (SVHN) is obtained from Google. Each picture contains a group of Arabic numerals '0–9′, which contains 73257 digits, and the image pixel is 32 × 32.  Figure 3 shows examples of USPS, MNIST, and SVHN. We can see that the distributions of USPS and MNIST are different but they contribute the same feature space. SVHN datasets are different in their distribution and feature space. We extract 9000 images from MNIST and SVHN as two domains. Since USPS has only 9298 pictures, we regard the whole dataset as a domain.

#### 4.1.2. Experimental Data

In this part, we compare some single-source transfer learning algorithms and multisource transfer learning algorithms such as DCTN and MFSAN with our algorithm MTLBDA

**Figure 4:**
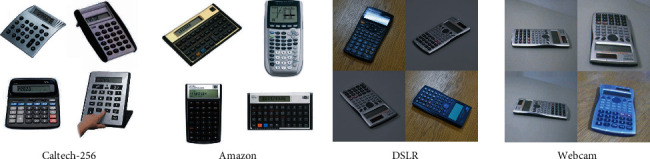
Example of Office-31 and Caltech-256 pictures.

It can be seen from Table 2 that among the three cross-domain tasks, the highest accuracy rates of the MTLBDA algorithm are 83.56%, 98.43%, and 96.14%, which are higher by 3.31%, 0.35%, and 0.02% than the algorithms of DCTN, MFSAN, and M^3^SDA, respectively. Compared with the classification tasks with S as the target domain or source domain, the multisource transfer learning algorithm is clearly better than the single-source transfer learning algorithm. Due to the single-source transfer learning algorithm, in the tasks with S as the target domain, our algorithm MTLBDA is 13.04% more accurate than the best transfer learning algorithm DDAN. From Figure 4, we can see that the different types of S, U, and *M* pictures lead to their distribution differences, which also proves the accuracy of our algorithm.

### 4.2. Image Classification Dataset

#### 4.2.1. Dataset Introduction

The Office-31 dataset is a commonly used standard transfer learning dataset. It contains 4652 sample pictures collected from different areas, namely, Amazon (A), webcam (W), and DSLR (D), and these pictures can be divided into 31 categories. Among them, Amazon's samples are from https//www.amazon.com, and the samples in webcam and DSLR are obtained through web cameras and digital SLR cameras in different environments. Caltech-256 [38] is a standard database for object recognition. The database has 30607 images and 256 categories. In these experiments, we used the dataset as Office-31+Caltech published by Gong [37] et al., as shown in Figure 4. Specifically, we have four domains, H (Caltech-256), A (Amazon), W (webcam), and D (DSLR). We randomly select three domains as the source domain and the remaining one as the target domain, that is, (A,H,D- > W), (A,H,W- > D), (A,D,W- > H), and (H,D,W- > A).

#### 4.2.2. Experimental Data

In this part, we compare some single-source transfer learning algorithms and multisource transfer learning algorithms such as DCTN and MFSAN with our algorithm MTLBDA.

It can be seen from Table 3 that among the four cross-domain tasks, the highest accuracy of the MTLBDA algorithm is 93.03%, 99.28%, 99.52%, and 94.53%, which are higher than those of the comparative algorithms DCTN, MFSAN, and M^3^SDA. At the same time, compared with the four cross-domain tasks, the single-source transfer learning algorithm shows higher accuracy than the optimal classification task by 4.25%, 0.82%, 3.16%, and 2.77%. In the A,W,D - > H task, MFSAN improved by 1.6% compared with the best transfer learning algorithm and by 3.25% compared with the best single-source learning algorithm. In contrast, the average accuracy is greatly improved, which proves the effectiveness of the proposed algorithm.

### 4.3. Influence of Category and *μ*

**Figure 5:**
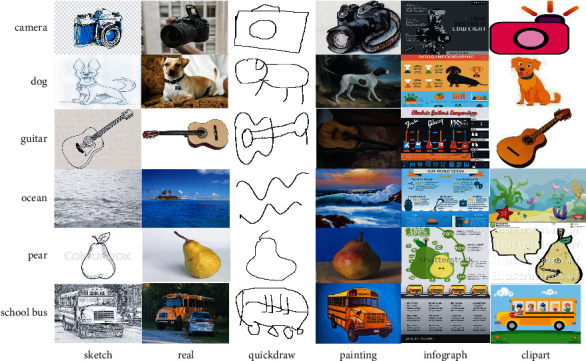
Example of DomainNet pictures.

To demonstrate the advantages of our algorithm category, we selected the network dataset domain proposed in Ref. [27] (as shown in Figure 5); the fields of the dataset are clipart, infographic, painting, quickdraw, real, and sketch, including 345 classes and 599859 data. The data distribution is shown in Table 4. Each domain contains 345 classes. We gradually increase the number of classes from 20 to 345 and show the impact of the number of iterations on the accuracy of the algorithm. Finally, we calculate the sensitivity of the algorithm to *μ*.

#### 4.3.1. Experimental Data

**Figure 6:**
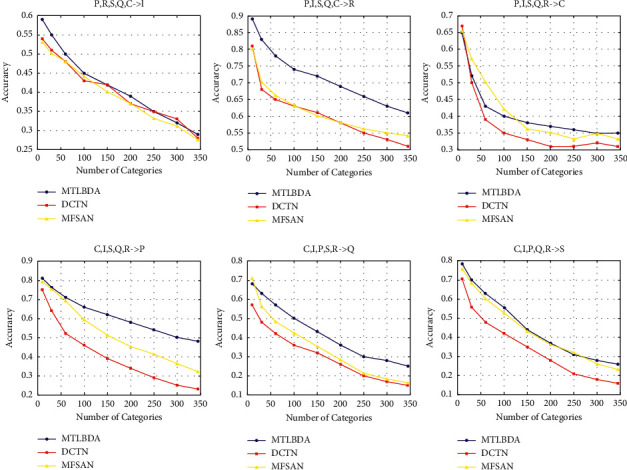
Comparison on DomainNet.

*(1) Category Influence*. We plot how the performances of different models will change when the number of categories increases. The figure shows all multidomain combinations. Under DomainNet, it can be seen from Figure 6(a)) that the multisource transfer learning algorithm is very sensitive to the number of classes. At the same time, when there are many classes, our algorithm is clearly better than the algorithms DCTN and MFSAN. (b) When the number of classes is greater than 150, our algorithm's accuracy is generally higher than that of other algorithms. (c) Our algorithm has a better effect on datasets with a very large difference between edge distribution and conditional probability distribution, such as DomainNet, which also proves that marginal probability and conditional probability have a great impact on classification in practical images.

**Figure 7:**
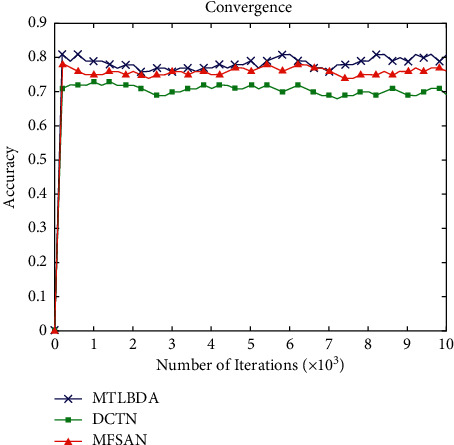
Effect of iteration time on accuracy.

*(2) Influence of Iterations*.  Figure 7 shows the effect of the number of iterations on the accuracy. (a) When the number of iterations exceeds 1000, the accuracy of the algorithm tends to be stable. (b) At the same time, MTLBDA shows better results.

**Figure 8:**
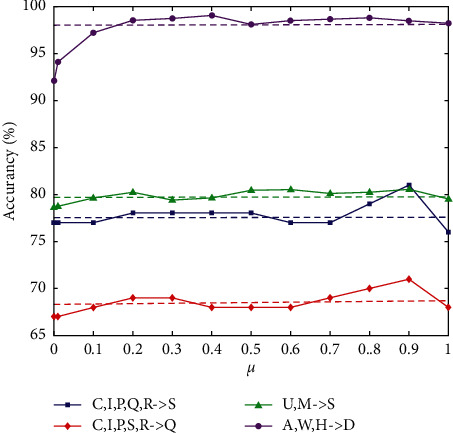
Effect of *μ* on accuracy.

*(3) Influence of μ*. In this section, we will evaluate the effectiveness of the balance factor µ. We used running *μ* ∈ {0,0.1,…, 0.9, 1.0} in MTLBDA with *μ*=0.5 as the baseline on some tasks.  Figure 8 shows the results. Clearly, the optimal *µ* is different in different tasks, indicating the importance of balancing the marginal distribution and conditional distribution between domains. In tasks C, I, P, *Q*, R- > S and C, I, P, S, *R* - > *Q* with optimal μ=0.9, the marginal distribution is almost the same, so the performance of transfer learning mainly depends on the conditional distribution. In task U, *M* - > S with optimal μ=0.4, the contribution of marginal distribution and conditional distribution is almost the same, but the marginal probability is more important. The observations were similar in other tasks. This shows that µ is essential for balancing marginal distribution and conditional distribution in cross-domain learning problems. Therefore, MTLBDA is more capable of obtaining good performance.

**Figure 9:**
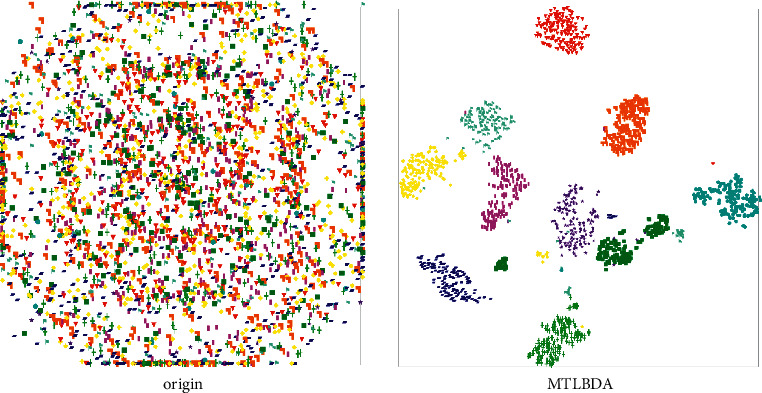
T-SNE plot of Caltech.

*(4) Feature Visualization*. From Figure 9, we can get the t-SNE plot of MTLBDA. Compared with the original state, we find that MTLBDA's clustering of features is very compact, which indicates MTLBDA-learned features have very ideal discrimination characteristics.

## 5. Conclusion

In this article, to solve the problem of unbalanced categories in multiple source fields in transfer learning, a small sample data classification technique based on category adaptation balance and multisource transfer learning is proposed. Under the condition of unbalanced distribution, this method first maps multiple source domains and target domains to the same target space. Then, according to the balanced distribution adaption algorithm, the distribution in each source domain and target domain is balanced while adjusting its marginal distribution and conditional distribution. Then the convolutional neural network is used as the classifier for each source domain and target domain. Finally, the regularization term of each source domain is added to prevent overfitting of the model. The experimental results on the SVHN dataset, USPS dataset, MNIST dataset, Office-31 dataset, Caltech-256 dataset, and DomainNet dataset show that MTLBDA is superior to the benchmark algorithm in classification accuracy and training efficiency. Although the experimental results show that the MTLBDA algorithm is better than the benchmark algorithm, in the future, further research is still needed in the following area: the expansion of MTLBDA to the multiclassification problem; the accurate estimation of *μ* is also a challenge.

## Figures and Tables

**Table 1:** MTLBDA algorithm steps.

MTLBDA algorithm training
Input: N source domains *X*^*s*^={*X*^*s*_*i*_^={(*x*_*j*_^*S*_*i*_^, *y*_*j*_^*S*_*i*_^)}_*j*=1_^*N*_*s*_*i*__^ · *i*=1, ..., *N*}. The number of label samples in each source domain is *N*_*S*_*i*__；the target domain is *X*^*t*^={(*x*_*j*_^*t*^)}_*j*=1_^*N*_*t*_^.
Output: Loss function *f*(*x*)
1: Give the number of training iterations *T* 2: From 1 to *T* 3: Randomly take *m* samples from a certain source domain 4: Take *m* samples from the target domain 5: Send the source and target samples to a common feature extractor, and get a common expression as *f*(·) 6: Input the common latent representation of the source sample into the domain-specific distribution balancer to obtain the domain-specific representation of the source sample *b*(·) 7: The specific domain representation of the original sample is output to the specific domain classifier, and the calculation formula of the classifier is (3) 8: The general latent representation of the target sample is input to all domain-specific extractors to obtain the domain-specific representation of the target sample. 9: Use the formula to calculate balance loss (7) 10: Make all passes to minimize the total loss in formula (10), update public feature extractor *F*(·)、multiple domain distribution balancer *B*_1_ *B*_2_ ⋯ *B*_*N*_ and multiple classifiers *C*_1_ *C*_2_ ⋯ *C*_*N*_, multiple regularization terms *R*_1_ *R*_2_ ⋯ *R*_*N*_。 11: Finish

**Table 2:** Average classification accuracy (%) on the digital classification datasets.

Algorithms	U- > S	M- > S	U- > M	S- > M	M- > U	S- > U
DAN	68.23	67.84	97.5	66.91	93.49	65.33
	(0.43)	(0.41)	(0.62)	(0.83)	(0.85)	(1.12)
DANN	68.65	68.14	97.92	67.23	93.47	66.25
	(0.88)	(0.82)	(0.81)	(0.94)	(0.79)	(0.97)
BDA	68.36	67.72	98.13	67.54	93.62	65.13
	(0.45)	(0.39)	(0.41)	(0.73)	(0.51)	(0.82)
DDAN	70.52	69.53	98.22	68.95	94.23	66.89
	(0.61)	(0.59)	(0.46)	(0.39)	(0.41)	(0.55)

	U, M- > s		U, S- > m		M, S- > u	

DCTN	77.61	76.83	96.23	96.85	92.81	93.08
	(0.41)	(0.39)	(0.82)	(0.73)	(0.47)	(0.56)
MFSAN	78.56	78.16	98.08	97.78	94.23	93.83
	(0.95)	(1.12)	(0.97)	(1.17)	(0.79)	(0.92)
M3SDA	80.25	79.47	97.48	97.63	94.67	95.31
	(0.82)	(0.75)	(0.85)	(0.91)	(0.93)	(0.96)
MTLBDA	83.56	82.82	97.91	98.43	96.14	94.81
	(0.66)	(0.41)	(0.76)	(0.68)	(0.81)	(1.03)

**Table 3:** Average classification accuracy (%) on the image classification datasets.

Algorithms	A- > H	W- > H	D- > H	A- > D	W- > D	H- > D
DAN	81.73	70.87	77.96	95.71	98.25	97.12
	(0.89)	(1.35)	(0.77)	(0.59)	(0.55)	(0.42)
DANN	85.23	75.31	83.15	96.12	98.12	97.35
	(1.06)	(1.12)	(0.93)	(0.87)	(0.83)	(0.78)
BDA	87.95	80.47	85.73	95.42	98.46	97.52
	(0.66)	(0.78)	(0.57)	(0.64)	(0.56)	(0.55)
DDAN	89.34	80.22	86.45	96.83	98.79	98.17
	(0.94)	(0.97)	(1.04)	(0.98)	(0.93)	(1.03)

	A- > W	D- > W	H- > W	W- > A	D- > A	H- > A

DAN	93.47	96.31	93.69	88.84	90.27	90.82
	(0.87)	(0.42)	(0.76)	(1.08)	(1.12)	(1.23)
DANN	95.31	96.24	95.75	89.25	91.76	90.41
	(0.82)	(0.45)	(1.01)	(1.06)	(1.18)	(1.07)
BDA	96.24	96.14	95.43	90.55	91.43	90.73
	(0.62)	(0.53)	(0.75)	(0.87)	(0.85)	(0.74)
DDAN	96.52	96.84	96.26	90.95	92.13	91.44
	(1.01)	(0.99)	(0.87)	(0.82)	(0.86)	(0.99)

	A, W, D- > H			A, W, H- > D		

DCTN	89.51	90.24	88.65	98.25	99.06	98.76
	(0.53)	(0.48)	(0.71)	(0.45)	(0.52)	(0.47)
MFSAN	91.43	90.54	91.17	99.27	98.03	98.77
	(0.48)	(0.69)	(0.56)	(0.58)	(0.65)	(0.52)
M^3^SDA	91.22	90.63	90.58	98.96	98.48	98.65
	(0.52)	(0.49)	(0.54)	(0.63)	(0.46)	(0.43)
MTLBDA	92.24	91.89	92.03	99.01	99.28	98.68
	(0.35)	(0.42)	(0.43)	(0.45)	(0.51)	(0.49)

	A, D, H- > W			W, D, H- > A		

DCTN	97.67	98.82	99.03	92.71	90.37	91.63
	(0.65)	(0.57)	(0.55)	(0.67)	(0.85)	(0.76)
MFSAN	99.48	98.37	99.08	91.54	93.26	94.14
	(0.38)	(0.69)	(0.43)	(0.75)	(0.82)	(0.65)
M^3^SDA	99.31	99.15	98.78	93.72	92.63	94.26
	(0.48)	(0.51)	(0.62)	(0.68)	(0.59)	(0.63)
MTLBDA	99.52	98.63	98.75	94.53	92.91	93.86
	(0.47)	(0.54)	(0.61)	(0.55)	(0.72)	(0.53)

**Table 4:** Data distribution example of DomainNet.

Domain	Category number	Total of data	Number of data of each subcategory (example)		
			Furniture	Mammal	Tool
Clipart	345	48921	5802	3437	3812
Infographic	345	53779	6513	3602	3096
Painting	345	76794	5002	8982	5124
Quickdraw	345	173500	17500	12500	14000
Real	345	70465	17104	15538	12938
Sketch	345	599859	7529	5151	4876

## Data Availability

The article uses four datasets: the USPS dataset, MNIST dataset, Office-31 dataset, and Caltech dataset, which can be downloaded from the Internet. MNIST: http://yann.lescun.com/exdb/mnist/. USPS: https://www.csie.ntu.edu.tw/∼cjlin/libsvmtools/datasets/multiclass.html#usps. Office-31: https://www.cc.gatech.edu/∼judy/domainadapt/#datasets_code. Caltech: http://www.vision.caltech.edu/Image_Datasets/Caltech256/.

## References

[1] Pan S. J., Yang Q. (2010). A survey on transfer learning. *IEEE Transactions on Knowledge and Data Engineering*.

[2] Weiss K., Khoshgoftaar T. M., Wang D. (2016). A survey of transfer learning. *Journal of Big Data*.

[3] Long M., Han Z., Wang J., Michael I., Jordan Deep transfer learning with joint adaptation networks.

[4] Tzeng E., Hoffman J., Zhang N., Saenko K., Trevor Darrell Deep domain confusion: maximizing for domain invariance.

[5] Long M., Cao Y., Wang J., Jordan M., Bach F., Blei D. (2015). Learning transferable features with deep adaptation networks. *Proceedings of the 32nd International Conference on Machine Learning, Volume 37 of Proceedings of Machine Learning Research*.

[6] Sun B., Feng J., Saenko K. (2016). Return of frustratingly easy domain adaptation. *AAAI*.

[7] Peng X., Saenko K. Synthetic to real adaptation with generative correlation alignment networks.

[8] Khan M. N. A., Heisterkamp D. R. Adapting instance weights for unsupervised dom ain adaptation using quadratic mutual information and subspace learning.

[9] Zadrozny B. Learning and evalua ting classifiers under sample selection bias.

[10] Cortes C., Mohri M., Riley M., Rostamizadeh A. (2008). Sample selection bias correction theory. *Lecture Notes in Computer Science, Ternational Conference on Algo-Rithmic Learning Theory*.

[11] Dai W., Yang Q., Xue G.-R., Y u Y. Boosting for transfer learning.

[12] Long M., Cao Y., Wang J., Jordan M. Learning transferable features with deep adaptation networks.

[13] Ganin Y., Lempitsky V., Bach F., Blei D. Unsupervised domain adaptation by backpropagation.

[14] Tzeng E., Hoffman J., Saenko K., Trevor Darrell Adversarial discriminative domain adaptation.

[15] Long M., Wang J., Ding G., Sun J., Yu P. S. Transfer Feature Learning with Joint Distribution Adaptation.

[16] Hou C.-A., Tsai Y.-H. H., Yeh Y.-R., Wang Y.-C. F. (2016). Unsupervised domain adaptation with label and structural consistency. *IEEE Transactions on Image Processing*.

[17] Tahmoresnezhad J., Hashemi S. (2016). Visual Domain Adaptation via Transfer Feature Learning. *Knowl. Inf. Syst*.

[18] Tzeng E., Hoffman J., Zhang N., Kate S Deep Domain Confusion: Maximizing for Domain Invariance.

[19] Zhu Y., Zhuang F., Wang J. (2020). Deep subdomain adaptation network for image classification. *IEEE Transactions on Neural Networks and Learning Systems*.

[20] Wang J., Chen Y., Feng W., Yu H., Huang M., Yang Q. (2020). Transfer learning with dynamic distribution adaptation. *ACM Transactions on Intelligent Systems and Technology*.

[21] Zhao H., Zhang S., Wu G., Moura J. M., Costeira J. P., Gordon G. J. (2018). Adversarial multiple source domian adpation. *NeuIPS*.

[22] Xu R., Chen Z., Zuo W., Yan J, Liang L Deep cocktail network: multi-source unsupervised domain adaptation with category shift.

[23] Hou C.-A., Yeh Y.-R., Wang Y.-C. F. An unsupervised domain adaptation approach for cross-domain visual classification.

[24] Crammer K., Kearns M., Wortman J. (2008). Learning from multiple sources. *Journal of Machine Learning Research*.

[25] Mansour Y., Mohri M., Rostamizadeh A. (2009). Domain adaptation with multiple sources. *Advances in Neural Information Processing Systems*.

[26] Ben-David S., Blitzer J., Crammer K., Kulesza A., Pereira F., Vaughan J. W. (2010). A theory of learning from different domains. *Machine Learning*.

[27] Peng X., Bai Q., Xia X. Moment Matching for Multi-Source Domain Adaptation.

[28] Zhu Y., Zhuang F., Wang D. (2019). Aligning domain-specific distribution and classifier for cross-domain classification from multiple sources. *Proceedings of the AAAI Conference on Artificial Intelligence*.

[29] Gretton A., Borgwardt K. M., Rasch M. J., Sch¨olkopf B., Smola A. (2012). A kernel two-sample test. *JMLR*.

[30] Sun B., Feng J., Saenko K. (2016a). Return of frustratingly easy domain adaptation. *AAAI*.

[31] Gretton A., Borgwardt K. M., Rasch M., Sch¨olkopf B., Smola A. J. (2007). A kernel method for the two-sample-problem. *Advances in Neural Information Processing Systems*.

[32] Zhao H., combes R. T. d, Zhang K., Gordon G. J. On learning invariant representation for domain adaptation.

[33] Pan S. J., Tsang I. W., Kwok J. T., Yang Q. (2011). Domain adaptation via transfer component analysis. *IEEE Transactions on Neural Networks*.

[34] Li X., Xiong H., Wang H. DELTA: DEEP LEARNING TRANSFER USING FEATURE MAP WITH ATTENTION FOR CONVOLUTIONAL NET- WORKS.

[35] Netzer Y., Wang T., Coates A., Bissacco A., Wu B., Ng A. Y. (2011). Reading Digits in Natural Images with Unsupervised Feature Learning.

[36] Li X., Fang M., Zhang J.-J. (2015). Projected Transfer Sparse Coding for cross domain image representation. *Journal of Visual Communication and Image Representation*.

[37] Long M., Wang J., Ding G., Pan S. J., Yu P. S. (2014). Adaptation regularization: a general framework for transfer learning. *IEEE Transactions on Knowledge and Data Engineering*.

[38] Gong B., Shi Y., Sha F., Grauman K. Geodesic flow kernel for unsupervised domain adaptation.

[39] Gao P., Li J., Ding C. (2022). Multisource mobile transfer learning algorithm based on dynamic model compression. *Security and Communication Networks*.

[40] Gao P., Li J. (2021). Multi-source fast transfer learning algorithm base on support vector machine. *Applied Intelligence*.

